# Effects of orthokeratology with different back optic zone diameters on corneal biomechanics and myopia control: a 1-year randomized, double-blind, self-controlled study

**DOI:** 10.1186/s40662-026-00503-2

**Published:** 2026-07-21

**Authors:** Ruisi Xie, Yan Huo, Xiaoqin Chen, Xiaorui Liu, Ying Guo, Zhengyuan Qu, Yutong Li, Xiaoyu Zhang, Haohan Zou, Guoxing Zhao, Yan Wang

**Affiliations:** 1 School of Medicine, Nankai University, Tianjin, 300071 China; 2 Tianjin Key Lab of Ophthalmology and Visual Science, Tianjin Eye Institute, Tianjin Eye Hospital, Nankai University Affiliated Eye Hospital, No. 4, Gansu Road, Heping District, Tianjin, 300020 China; 3 Tianjin Eye Hospital Optometric Center, Tianjin, 300041 China; 4 Clinical College of Ophthalmology, Tianjin Medical University, Tianjin, 300070 China; 5 Nankai University Eye Institute, Nankai University, Tianjin, 300071 China

**Keywords:** Myopia control, Corneal biomechanics, Orthokeratology, Back optic zone diameter, Randomized controlled trial

## Abstract

**Background:**

To assess the long-term effects of orthokeratology (ortho-k) lenses with varying back optic zone diameters (BOZDs) on corneal biomechanical properties and explore their relationship with myopia control.

**Methods:**

This 1-year prospective, randomized, double-blind, self-controlled study enrolled 36 myopic children aged 9–12 years. Thirty subjects (60 eyes) completed the 1-year follow-up period. The subjects were randomly fitted with a 5-mm BOZD (5BOZD) lens in one eye and a 6-mm BOZD (6BOZD) lens in the fellow eye. Corneal biomechanics were measured using Corvis ST at baseline and at 1 day, 7 days, 1 month, 6 months, and 12 months after ortho-k wear. Axial length (AL) was measured at baseline, 6 months, and 12 months after ortho-k wear, and AL elongation (ΔAL) was calculated relative to baseline. Statistical analyses were performed to compare intergroup differences and examine the correlations between ΔAL and changes in biomechanical parameters.

**Results:**

ΔAL was significantly smaller in the 5BOZD groups compared to the 6BOZD groups at both 6 and 12 months (*P* < 0.05). The stiffness parameter at first applanation (SP-A1) significantly increased at 6 months in both groups (*P* < 0.05). At 12 months, the first applanation time (A1 time) and integrated radius significantly increased, while the first applanation velocity (A1 velocity) and stress–strain index (SSI) significantly decreased in both groups (*P* < 0.05). In the 6BOZD group, ΔAL showed a significant positive correlation with ΔA1 time at 6 and 12 months (r_6m_ = 0.385, r_12m_ = 0.424, *P* < 0.05).

**Conclusions:**

Long-term ortho-k therapy increased SP-A1, A1 time, integrated radius, and decreased A1 velocity, reflecting enhanced corneal resistance and elasticity. Compared to the 5BOZD lens, the traditional 6BOZD lens demonstrated relatively greater corneal elasticity, possibly reducing myopia control efficacy. A smaller BOZD may offset these corneal biomechanical changes and improve the management of myopia.

*Trial registration* Chinese Clinical Trial Registry (ChiCTR2200061048).

**Supplementary Information:**

The online version contains supplementary material available at 10.1186/s40662-026-00503-2.

## Background

Myopia is a major global public health concern [1]. The increase in its prevalence has led to a high risk of vision-threatening complications, including myopic maculopathy, retinal detachment, and glaucoma, which together account for approximately 10%–30% of global visual impairment and blindness [2]. Therefore, effective interventions are necessary to slow the progression of myopia. Orthokeratology (ortho-k) is a well-established clinical approach for myopia control and has been demonstrated to reduce axial length (AL) elongation by approximately 43%–63% [3–7]. This technique uses rigid gas-permeable contact lenses with reverse geometry to induce mechanical interactions with the cornea, thereby triggering corneal reshaping [8]. This can result in central corneal flattening and peripheral corneal steepening. These corneal changes temporarily correct refractive errors and improve uncorrected visual acuity [9]. However, the clinical efficacy of ortho-k in slowing axial elongation varies with lens design [10, 11].

Recent evidence suggests that ortho-k lenses with smaller back optic zone diameters (BOZDs) are more effective in myopia control [12], although the underlying mechanisms remain unclear. These lenses primarily induce mid-peripheral corneal steepening closer to the central cornea [13], thereby enhancing the optical signals related to myopia control, such as higher-order aberrations and peripheral myopic defocus [14, 15]. Nonetheless, the corneal reshaping response to ortho-k differs among individuals and cannot be fully explained using optical factors alone [16]. Zhang et al. [17] proposed that the interaction between ortho-k lenses and corneal biomechanics is fundamental to corneal morphological changes and the generation of optical signals that mediate myopia control. Compared to conventional designs, smaller BOZD lenses create a more concentrated mechanical treatment zone, leading to faster and greater corneal deformation [17]. This observation suggests that corneal biomechanics may be a potential mechanism underlying the myopia control efficacy of different BOZD designs. Moreover, the cornea exhibits viscoelastic properties, allowing it to maintain deformation for a certain period and subsequently recover when external mechanical stress is removed [18]. In addition, previous studies have demonstrated that corneal biomechanics change during ortho-k lens wear and that early alterations in biomechanical parameters, such as the stiffness parameter at first applanation (SP-A1) and the stress–strain index (SSI), could assist in predicting long-term changes in AL [19, 20]. However, no study has elucidated the intrinsic relationship between in vivo biomechanical factors and the myopia management efficacy of different BOZD ortho-k designs. Understanding the corneal biomechanical changes during ortho-k lens wear with different BOZD is crucial for exploring the mechanisms underlying myopia control and guiding personalized lens-fitting strategies.

Therefore, this study aimed to compare the differences in corneal biomechanical parameters and AL elongation between ortho-k lenses with 5-mm (5BOZD) and 6-mm BOZD (6BOZD) over a 1-year follow-up period, and to analyze the correlation between biomechanical changes and AL elongation. This lays the foundation for understanding the biomechanical mechanisms of myopia control and informs personalized ortho-k lens selection.

## Methods

This was a 1-year prospective, randomized, double-blind, self-controlled clinical study. Enrollment, baseline, and follow-up examinations were performed at the Tianjin Eye Hospital between June 2022 and December 2023. The study registration was recorded at Chictr.org.cn (ChiCTR2200061048). All protocols followed the Declaration of Helsinki. Informed consent was obtained from all the participants and their legal guardians.

### Participants and inclusion criteria

Low myopic children, age 9–12 years with cycloplegic spherical equivalent refraction between − 1.00 to − 4.00 diopters (D), astigmatism ≤ 1.50 D, anisometropia ≤ 1.00 D, and best-corrected monocular visual acuity (VA) of 0.0 logMAR (20/20) or better. The exclusion criteria were strabismus, abnormal binocular vision, contraindications for contact lens wear, and ocular or systemic diseases.

### Randomization and masking

After eligibility screening, two BOZD lens designs (5 mm and 6 mm) were randomly assigned to the two eyes of each participant. An independent statistician constructed a random number table using SPSS Statistics version 25.0 (IBM, Chicago, IL, USA). The randomization results were masked by the investigators responsible for screening and outcome assessment, as well as by technicians and participants to maintain double-masking.

### Ortho-k lens design and fitting

This study utilized corneal refractive therapy (CRT) ortho-k lenses composed of Paragon HDS100 material (Paragon Vision Sciences, Gilbert, AZ, USA). The CRT lens features a four-zone design, including a spherical base curve, a 1.0-mm wide sigmoid return zone, a tangential landing zone, and a peripheral zone. The remaining design parameters of the two ortho-K lenses, with the BOZD set at 5 mm or 6 mm, were identical. Experienced optometrists performed all lens fittings. The participants were instructed to wear ortho-k lenses for 8–10 h nightly and received comprehensive training on lens use and care. Follow-up assessments were performed at baseline and at 1-day, 7-day, 1-month, 6-month, and 12-month visits after lens wear initiation. Throughout the 12-month period, compliance was monitored using daily lens-wear diaries.

### Ophthalmic examinations and data collection

AL was measured at baseline, 6-month, and 12-month visits using IOL Master 500 (Carl Zeiss, Germany). Five consecutive measurements were obtained in each measurement session and were averaged. AL elongation was calculated as the difference between each follow-up and initial measurement.

Corneal biomechanics were measured using Corvis ST (Oculus, Wetzlar, Germany) at baseline, 1-day, 7-day, 1-month, 6-month, and 12-month visits. The equipment utilizes a Scheimpflug high-speed camera (4330 fps) to capture corneal deformation. The dynamic corneal response parameters and deformation waveforms were extracted for analysis. Based on previous studies [21, 22], we analyzed 14 biomechanical parameters using the Corvis ST software version 1.6b2042, as outlined in Additional file 1. All the examinations were performed by skilled ophthalmologists. Standard Operating Procedures on ortho-k lens assessment and ophthalmic examinations were used to minimize interobserver differences. Three measurements were obtained for each eye, and the mean values with a quality of “OK” were analyzed. The analyzed data originated from CSV files.

### Sample size calculation

The sample size was estimated using effect sizes from previous studies [14], with a significance level of 0.05, and a power of 0.8. A minimum of 30 participants was required per group. Accounting for an assumed 20% dropout rate over the 1-year study period, at least 36 participants were recruited per group.

### Statistical analysis

All analyses were performed using the SPSS Statistics version 25.0. Continuous data were presented as average ± standard deviation, while categorical data were displayed as numbers with percentages. The normality of the baseline ocular and corneal biomechanical data was evaluated using the Shapiro–Wilk test. Paired t-tests were used to compare baseline ocular characteristics and AL changes between the groups. Longitudinal biomechanical changes were analyzed using generalized estimating equations (GEEs) with exchangeable correlation structures and an identity linking function, along with robust standard errors, to account for within-subject correlations between eyes and incorporate all available data [23, 24]. The parameters provided by the GEEs were the effect size and standard error (SE). After adjusting for potential covariates, including central corneal thickness (CCT) and intraocular pressure (IOP), GEEs were used to evaluate the longitudinal changes in corneal biomechanical parameters and group differences during follow-up. *P* values for pairwise comparisons were adjusted for multiple comparisons using a sequential Bonferroni correction. The association between AL elongation and biomechanical changes was assessed using a partial correlation analysis after adjusting for CCT and IOP. A *P* value of 0.05 denotes statistical significance.

## Results

### Baseline characteristics

**Fig. 1:**
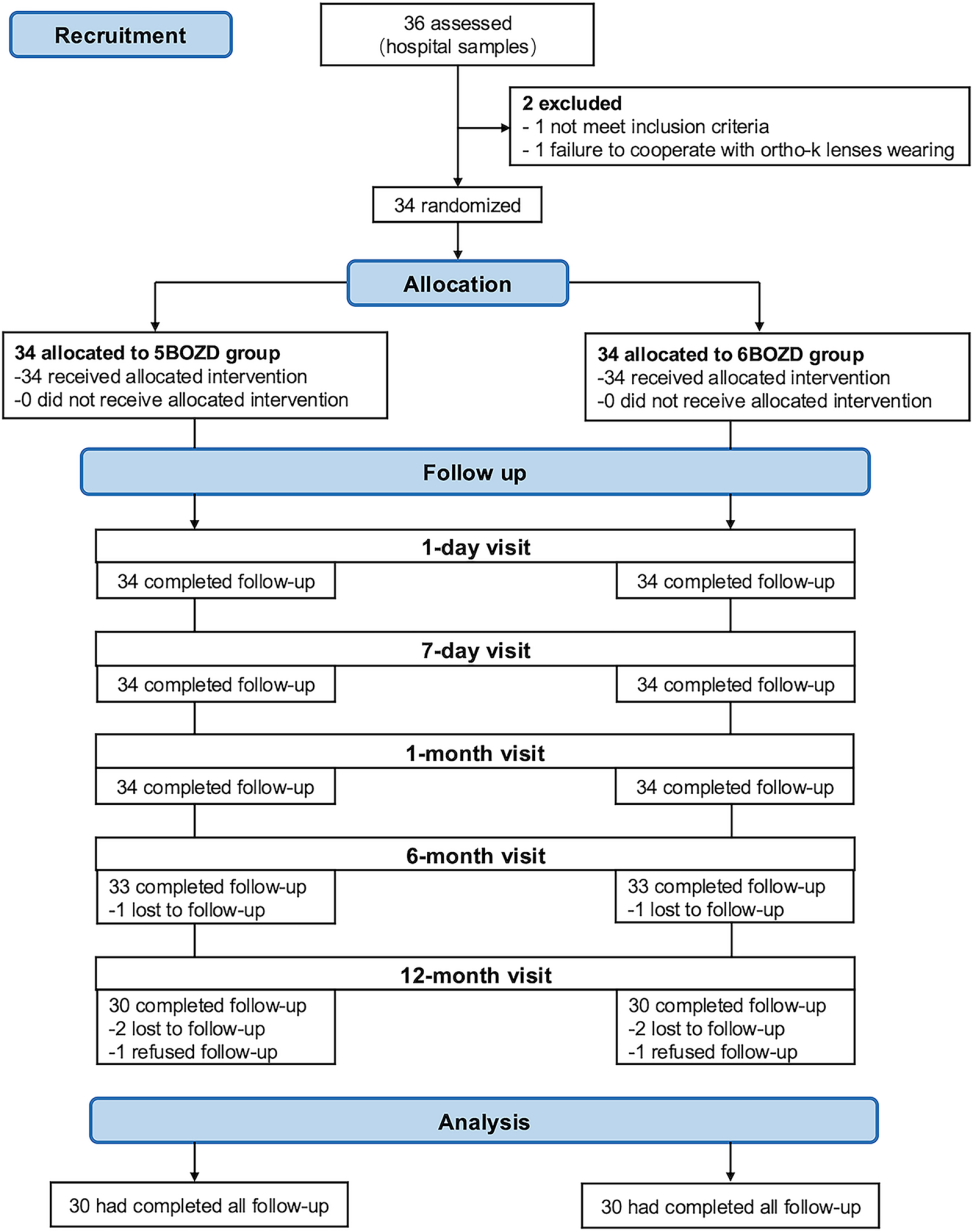
Consolidated standards of reporting trials (CONSORT) of the study

A total of 36 participants were recruited for this study. Following the exclusion of one participant who failed to meet the eligibility requirements, one participant who failed to use ortho-k lenses, and four participants who discontinued follow-up, 60 eyes of 30 participants (83.3%) were included in the final analysis (Fig. 1). No complications such as corneal abrasion, staining, or infiltrative events were observed throughout the study. The baseline ocular characteristics and AL changes were normally distributed. Table 1 shows that no significant variations in the initial spherical equivalent refraction (SER), flattest keratometry (Kf), steepest keratometry (Ks), AL, CCT, or IOP were observed between the 5BOZD and 6BOZD groups (*P* > 0.05).

**Table 1:** Ocular baseline information of participants

Characteristics	5BOZD	6BOZD	*P* value
Sphere (D)	− 2.47 ± 0.77	− 2.48 ± 0.88	0.941
SER (D)	− 2.83 ± 0.82	− 2.84 ± 0.87	0.896
Kf (D)	42.94 ± 1.59	42.89 ± 1.57	0.288
Ks (D)	44.38 ± 1.67	44.37 ± 1.71	0.909
AL (mm)	24.51 ± 0.92	24.52 ± 0.91	0.848
IOP (mmHg)	16.25 ± 1.68	15.75 ± 2.02	0.110
CCT (μm)	555.63 ± 31.82	557.87 ± 30.56	0.188

Data are shown as mean ± standard deviation. *AL =* axial length; *BOZD* = back optic zone diameter; *CCT* = central corneal thickness; *IOP* = intraocular pressure; *Kf* = flattest keratometry; *Ks* = steepest keratometry; *SER* = spherical equivalent refractive

### Temporal evolution of corneal biomechanical parameters

The changes in biomechanical parameters in the 5BOZD and 6BOZD groups at baseline and during the 1-year follow-up period are shown in Table 2. After accounting for confounding variables (CCT and IOP) based on correlation analyses [23], no significant intergroup differences were observed in the biomechanical variables at baseline or at any follow-up point (*P* > 0.05).

**Table 2:** Comparison of biomechanical parameters between the 5BOZD and 6BOZD groups during ortho-k treatment

Parameters		Pre	Post 1 D	Post 7 D	Post 1 M	Post 6 M	Post 12 M	*P* value
Def. amp. max	5BOZD	1.098 ± 0.008	1.102 ± 0.012	1.089 ± 0.013	1.073 ± 0.012	1.088 ± 0.022	1.095 ± 0.013	^1^*P* > 0.05 ^2^*P* = 0.025^c^ ^3^*P* < 0.05^c,d^
	6BOZD	1.103 ± 0.012	1.105 ± 0.013	1.088 ± 0.017	1.070 ± 0.013	1.066 ± 0.011	1.093 ± 0.014	
A1 time	5BOZD	7.380 ± 0.006	7.390 ± 0.006	7.386 ± 0.006	7.385 ± 0.005	7.394 ± 0.005	7.392 ± 0.005	^1^*P* > 0.05 ^2^*P* < 0.05^d^^,^^e^ ^3^*P* = 0.031^e^
	6BOZD	7.376 ± 0.006	7.375 ± 0.006	7.368 ± 0.006	7.380 ± 0.006	7.388 ± 0.005	7.393 ± 0.005	
A1 velocity	5BOZD	0.152 ± 0.001	0.150 ± 0.002	0.146 ± 0.002	0.147 ± 0.002	0.149 ± 0.002	0.147 ± 0.002	^1^*P* > 0.05 ^2^*P* < 0.05^b,c,e^ ^3^*P* < 0.05^c–e^
	6BOZD	0.153 ± 0.002	0.151 ± 0.002	0.150 ± 0.003	0.145 ± 0.002	0.148 ± 0.002	0.145 ± 0.002	
A2 time	5BOZD	22.086 ± 0.051	22.040 ± 0.087	22.121 ± 0.035	22.115 ± 0.028	22.039 ± 0.112	22.150 ± 0.037	^1^*P* > 0.05 ^2^*P* > 0.05 ^3^*P* > 0.05
	6BOZD	22.032 ± 0.126	22.050 ± 0.072	22.170 ± 0.048	22.128 ± 0.039	22.099 ± 0.045	22.208 ± 0.030	
A2 velocity	5BOZD	− 0.281 ± 0.006	− 0.286 ± 0.008	− 0.279 ± 0.004	− 0.279 ± 0.004	− 0.269 ± 0.012	− 0.287 ± 0.005	^1^*P* > 0.05 ^2^*P* > 0.05 ^3^*P* = 0.046^b^
	6BOZD	− 0.278 ± 0.007	− 0.281 ± 0.006	− 0.292 ± 0.006	− 0.283 ± 0.005	− 0.271 ± 0.005	− 0.281 ± 0.005	
HC time	5BOZD	17.233 ± 0.092	17.110 ± 0.075	17.205 ± 0.094	17.158 ± 0.068	17.238 ± 0.091	17.090 ± 0.097	^1^*P* > 0.05 ^2^*P* > 0.05 ^3^*P* > 0.05
	6BOZD	17.223 ± 0.089	17.183 ± 0.102	17.134 ± 0.112	17.296 ± 0.076	17.272 ± 0.099	17.126 ± 0.083	
Peak dist	5BOZD	5.068 ± 0.028	5.052 ± 0.033	5.005 ± 0.032	4.985 ± 0.034	5.016 ± 0.035	5.036 ± 0.031	^1^*P* > 0.05 ^2^*P* < 0.05^b, c^ ^3^*P* = 0.025^d^
	6BOZD	5.056 ± 0.028	5.056 ± 0.031	5.070 ± 0.029	5.041 ± 0.030	4.986 ± 0.032	5.057 ± 0.032	
Radius	5BOZD	7.096 ± 0.119	7.008 ± 0.144	6.858 ± 0.138	6.836 ± 0.182	6.860 ± 0.164	6.820 ± 0.119	^1^*P* > 0.05 ^2^*P* > 0.05 ^3^*P* > 0.05
	6BOZD	7.151 ± 0.193	7.064 ± 0.181	6.996 ± 0.138	7.083 ± 0.117	7.046 ± 0.194	6.851 ± 0.147	
DA ratio max (1 mm)	5BOZD	1.568 ± 0.006	1.569 ± 0.006	1.563 ± 0.006	1.576 ± 0.006	1.574 ± 0.006	1.590 ± 0.021	^1^*P* > 0.05 ^2^*P* > 0.05 ^3^*P* > 0.05
	6BOZD	1.564 ± 0.006	1.572 ± 0.005	1.570 ± 0.005	1.573 ± 0.005	1.570 ± 0.005	1.570 ± 0.006	
DA ratio max (2 mm)	5BOZD	4.279 ± 0.066	4.294 ± 0.045	4.229 ± 0.055	4.267 ± 0.035	4.310 ± 0.040	4.395 ± 0.106	^1^*P* > 0.05 ^2^*P* > 0.05 ^3^*P* > 0.05
	6BOZD	4.363 ± 0.114	4.423 ± 0.149	4.311 ± 0.040	4.267 ± 0.051	4.246 ± 0.045	4.365 ± 0.095	
ARTh	5BOZD	494.38 ± 16.07	429.53 ± 14.48	380.85 ± 14.86	392.86 ± 20.40	393.38 ± 19.14	388.05 ± 16.65	^1^*P* > 0.05 ^2^*P* < 0.001^a–e^ ^3^*P* < 0.001^a–e^
	6BOZD	473.04 ± 17.38	405.30 ± 11.16	370.56 ± 12.88	358.73 ± 14.23	365.39 ± 14.90	371.24 ± 19.55	
Integrated radius	5BOZD	8.40 ± 0.14	8.49 ± 0.12	8.74 ± 0.14	8.74 ± 0.15	8.76 ± 0.17	8.81 ± 0.13	^1^*P* > 0.05 ^2^*P* < 0.05^e^ ^3^*P* = 0.01^e^
	6BOZD	8.42 ± 0.18	8.49 ± 0.12	8.71 ± 0.15	8.58 ± 0.12	8.63 ± 0.18	8.89 ± 0.15	
SP-A1	5BOZD	98.82 ± 1.36	102.90 ± 1.39	107.09 ± 1.59	106.68 ± 1.54	104.86 ± 1.38	101.66 ± 4.31	^1^*P* > 0.05 ^2^*P* < 0.05^a–d^ ^3^*P* < 0.05^a–e^
	6BOZD	97.42 ± 1.49	102.37 ± 1.26	106.01 ± 1.54	107.78 ± 1.33	106.14 ± 1.13	104.38 ± 1.39	
SSI	5BOZD	0.86 ± 0.01	0.84 ± 0.02	0.85 ± 0.02	0.86 ± 0.02	0.86 ± 0.03	0.83 ± 0.02	^1^*P* > 0.05 ^2^*P *< 0.032^e^ ^3^*P *< 0.05^d,^^e^
	6BOZD	0.87 ± 0.02	0.86 ± 0.02	0.83 ± 0.02	0.87 ± 0.02	0.91 ± 0.02	0.82 ± 0.02^e^	

*pre* = baseline; *post 1 D* = 1 day after ortho-k wear; *post 7 D* = 7 days after ortho-k wear; *post 1 M* = 1 month after ortho-k wear; *post 6 M* = 6 months after ortho-k wear; *post 12 M* = 12 months after ortho-k wear; *Def. Amp. Max* = the deformation amplitude at the highest concavity; *A1 time* = the first applanation time; *A1 velocity* = the first applanation velocity; *A2 time* = the second applanation time; *A2 velocity* = the second applanation velocity; *HC time =* the highest concavity time; *Peak Dist.* = peak distance; *DA Ratio Max 1 mm* = corneal deformation ratio between the corneal apex and corneal apex within 1 mm; *DA Ratio Max 2 mm* = corneal deformation ratio between the corneal apex and corneal apex within 2 mm; *ARTh* = Ambrósio relational thickness to the horizontal profile; *SP-A1* = the stiffness parameter at first applanation; *SSI* = stress–strain index

Data are shown as mean ± standard deviation. CCT and IOP were used as covariates

^1^*P* compares the 5 mm and 6 mm BOZD at baseline and each follow-up visit (1 day, 7 days, 1 month, 6 months, and 12 months)

^2^*P* compares baseline and each follow-up visit (1 day, 7 days, 1 month, 6 months, and 12 months) in the 5 mm BOZD group

^3^*P* compares baseline and each follow-up visit (1 day, 7 days, 1 month, 6 months, and 12 months) in the 6 mm BOZD group

^a^Statistically significant difference between baseline and 1-day visit

^b^Statistically significant difference between baseline and 7-day visit

^c^Statistically significant difference between baseline and 1-month visit

^d^Statistically significant difference between baseline and 6-month visit

^e^Statistically significant difference between baseline and 12-month visit

SP-A1 levels were significantly higher than baseline from 1-day to 6-month visits in both groups (*P* < 0.05). At 12 months, the SP-A1 level in the 6BOZD group remained significantly higher than that at baseline (*P* < 0.05), whereas no significant change was observed in the 5BOZD group. The integrated radius increased (*P* < 0.05), whereas the SSI decreased significantly at 12 months (*P* < 0.05).

The A1 velocity decreased significantly at the 7-day, 1-month, and 12-month visits in the 5BOZD group (*P* < 0.05) and at the 1-month, 6-month, and 12-month visits in the 6BOZD group (*P* < 0.05). The peak distance decreased significantly at 7 days and 1 month in the 5BOZD group and then stabilized (*P* < 0.05). In the 6BOZD group, it decreased at 6 months before reaching a plateau (*P* < 0.05). The deformation amplitude at the highest concavity (Def. Amp. Max) decreased significantly at 1 month in both groups (*P* < 0.05), remained stable in the 5BOZD group, and continued to decline through 6 months before stabilizing in the 6BOZD group.

The A1 time increased significantly at 6 months in the 5BOZD group and remained elevated at 12 months (*P* < 0.05). Similarly, the A1 time was significantly higher than that at baseline at 12 months in the 6BOZD group (*P* < 0.05). In contrast, the second applanation time (A2 time), the highest concavity time (HC time), radius, and the corneal deformation ratio between the corneal apex within 1 mm and 2 mm (DA ratio at 1 mm and 2 mm) did not show significant changes during follow-up (*P* > 0.05).

### Comparison of corneal biomechanical changes between groups

**Fig. 2:**
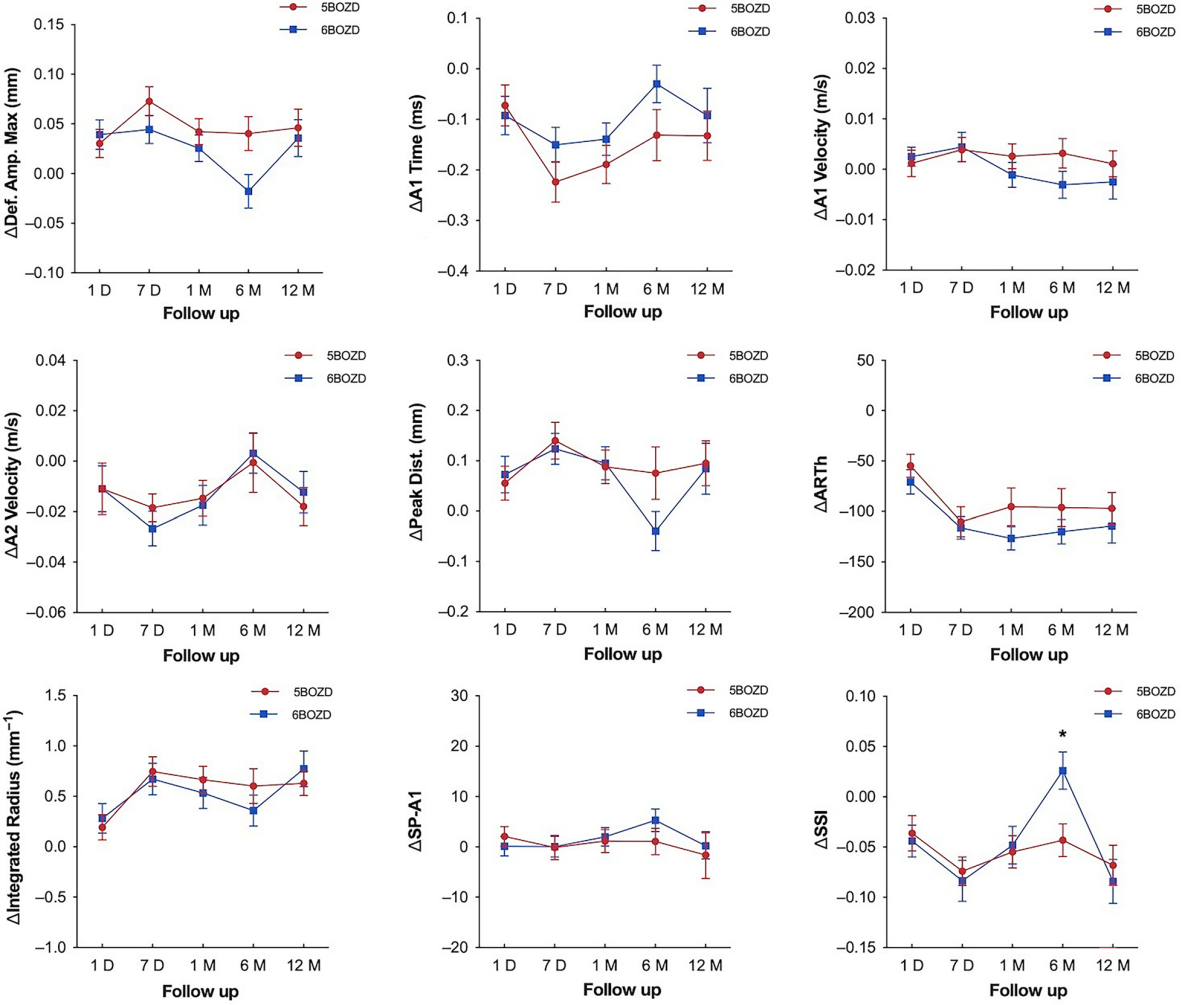
Follow-up trend of corneal biomechanical parameters during 1 year of orthokeratology (ortho-k) therapy. * P < 0.05. BOZD, back optic zone diameter; D, day; M, month; Def. Amp. Max, the deformation amplitude at the highest concavity; A1 time, the first applanation time; A1 velocity, the first applanation velocity; A2 velocity, the second applanation velocity; Peak Dist., peak distance; ARTh, Ambrósio relational thickness to the horizontal profile; SP-A1, the stiffness parameter at first applanation; SSI, stress–strain index

An intergroup comparison of biomechanical parameter changes over time is presented in Fig. 2. At 6 months, the change in SSI was significantly greater in the 6BOZD group than in the 5BOZD group (*P* = 0.004). No significant differences were observed in other corneal biomechanical parameters between the two groups at any time point during the 12-month follow-up period.

### AL elongation

**Fig. 3:**
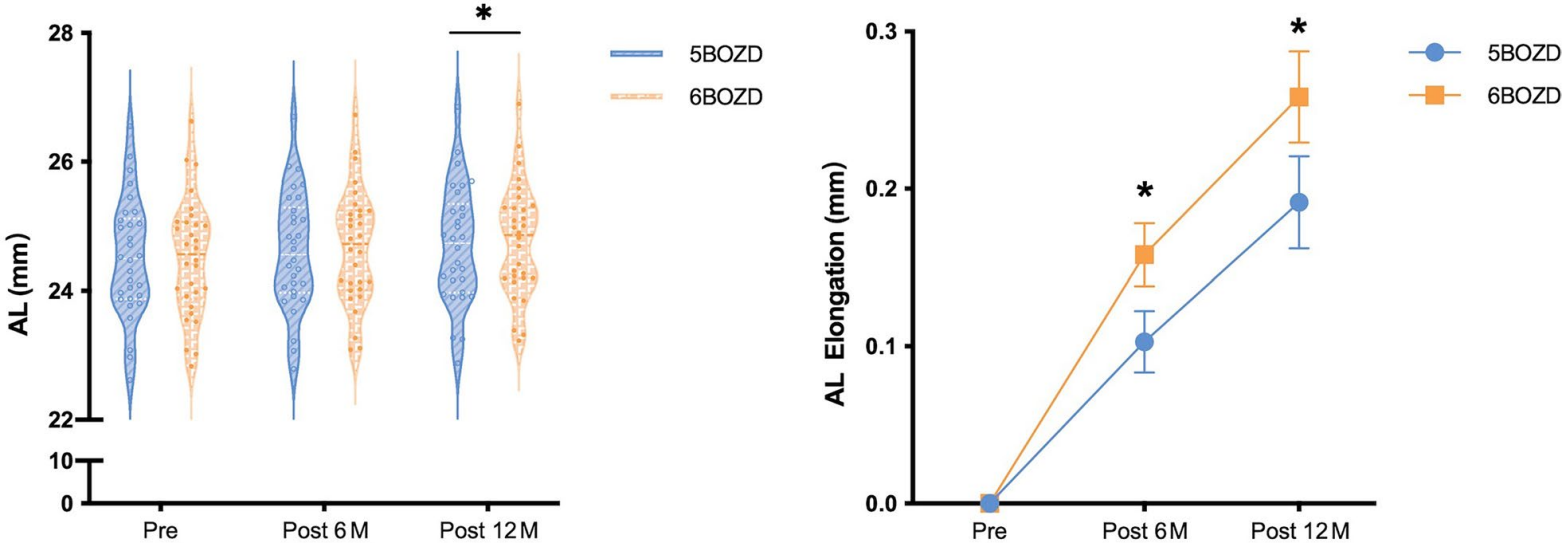
Comparison of axial length (AL) and AL elongation in the 5BOZD and 6BOZD groups. * *P* < 0.05. BOZD, back optic zone diameter; D, day; M, month

As shown in Fig. 3, At 6 months, the 5BOZD group had an average AL of 24.61 ± 0.91 mm, corresponding to an elongation of 0.103 ± 0.11 mm (Fig. 3), while the 6BOZD group showed a mean AL of 24.68 ± 0.90 mm, with an elongation of 0.158 ± 0.11 mm (Fig. 3). The increase in AL was significantly slower in the 5BOZD group than in the 6BOZD group (*P* = 0.001).

At 12 months, the mean AL was 24.70 ± 0.92 mm in the 5BOZD group and 24.78 ± 0.87 mm in the 6BOZD group, representing elongations of 0.191 ± 0.16 mm and 0.258 ± 0.16 mm, respectively. The increase in AL was significantly lower in the 5BOZD group (*P* = 0.004).

### Correlation between corneal biomechanical changes and AL elongation

After adjusting for CCT and IOP, correlation analyses were performed to assess the association between AL elongation and changes in corneal biomechanical parameters at 6 and 12 months (Table 3). In the 5BOZD group, there were no significant correlations between the changes in AL and any corneal biomechanical parameters at either 6 or 12 months (*P* > 0.05). In the 6BOZD group, the HC time (r = − 0.453) and ARTh (r = − 0.374) were significantly negatively correlated with AL elongation at 6 months (*P* < 0.05). Conversely, A1 time was significantly positively correlated with AL elongation at both 6 (r = 0.385) and 12 months (r = 0.424) (*P* < 0.05).

**Table 3:** Correlation between axial length (AL) elongation and change of biomechanical parameters in the 5BOZD and 6BOZD groups

Post 6 M − Pre	AL elongation			
	5BOZD		6BOZD	
	*r*	*P*	*r*	*P*
ΔDef. Amp. Max	0.063	0.747	− 0.215	0.263
ΔA1 time	0.254	0.183	0.385	**0.039**
ΔA1 velocity	− 0.184	0.339	0.112	0.565
ΔA2 time	0.022	0.908	− 0.303	0.104
ΔA2 velocity	− 0.054	0.775	0.220	0.251
ΔHC time	− 0.032	0.867	− 0.453	**0.014**
ΔPeak dist	0.072	0.714	− 0.062	0.754
ΔRadius	0.006	0.974	− 0.335	0.070
ΔDA ratio max (1 mm)	0.217	0.258	0.282	0.132
ΔDA ratio max (2 mm)	0.126	0.516	0.179	0.345
ΔARTh	− 0.169	0.381	− 0.374	**0.046**
ΔIntegrated radius	0.124	0.515	0.217	0.267
ΔSP-A1	0.071	0.713	− 0.047	0.807
ΔSSI	− 0.186	0.324	− 0.199	0.291
Post 12 M − Pre	AL elongation			
	5BOZD		6BOZD	
	r	*P*	r	*P*
ΔDef. amp. max	− 0.008	0.967	0.077	0.693
ΔA1 time	− 0.053	0.786	0.424	**0.022**
ΔA1 velocity	0.006	0.975	0.143	0.458
ΔA2 time	0.003	0.988	− 0.232	0.218
ΔA2 velocity	− 0.038	0.844	− 0.025	0.897
ΔHC time	0.051	0.789	− 0.113	0.553
ΔPeak dist	0.201	0.259	0.236	0.227
ΔRadius	0.089	0.642	− 0.241	0.200
ΔDA ratio max (1 mm)	0.284	0.135	0.304	0.109
ΔDA ratio max (2 mm)	0.147	0.437	0.009	0.963
ΔARTh	− 0.166	0.389	− 0.283	0.137
ΔIntegrated radius	0.211	0.281	0.184	0.339
ΔSP-A1	0.183	0.343	− 0.302	0.112
ΔSSI	0.003	0.986	− 0.292	0.131

*Δ* indicates the difference between baseline and 6-month or 12-month visits of ortho-k; *Post 6 M − Pre* indicates the difference between baseline and the 6-month visit; *Post 12 M − Pre* indicates the difference between baseline and the 12-month visit. *Def. Amp. Max* = the deformation amplitude at the highest concavity; *A1 time* = the first applanation time; *A1 velocity* = the first applanation velocity; *A2 time* = the second applanation time; *A2 velocity* = the second applanation velocity; *HC time* = the highest concavity time; *Peak dist.* = peak distance; *DA ratio max 1 mm* = corneal deformation ratio between the corneal apex and corneal apex within 1 mm; *DA ratio max 2 mm* = corneal deformation ratio between the corneal apex and corneal apex within 2 mm; *ARTh* = Ambrósio relational thickness to the horizontal profile; *SP-A1* = the stiffness parameter at first applanation; *SSI* = stress–strain index. *P* values in bold indicate statistical significance

## Discussion

To the best of our knowledge, this is the first randomized, self-controlled, double-blind clinical trial to evaluate sequential changes in corneal biomechanical parameters and AL over 1 year of 5BOZD and 6BOZD ortho-k treatment. There were no significant differences in baseline biomechanical parameters between the two groups, supporting the reliability of subsequent comparisons. After correcting for CCT and IOP, SP-A1 was significantly higher than at the initial visit in both groups at the 6-month visit (*P* < 0.05). The A1 time and integrated radius significantly increased, whereas the A1 velocity and SSI significantly reduced in both groups at the 12-month visit (*P* < 0.05). These findings suggest that long-term ortho-k treatment enhances corneal resistance to deformation and improves corneal plasticity. A significant positive correlation was found between changes in AL (ΔAL_6BOZD_) and A1 time (ΔA1 time) in the 6BOZD group (r_6m_ = 0.385, r_12m_ = 0.424; *P* < 0.05). This finding suggests that the relatively higher intrinsic change in corneal elasticity induced by the 6-mm BOZD ortho-k may underlie its reduced myopia control efficacy compared to the 5-mm design. This study provides objective evidence of corneal biomechanical changes during ortho-k wear and provides new insights into the mechanisms of myopia control and personalized lens fitting in clinical practice.

By adjusting for CCT and IOP, these two confounders were often overlooked in previous studies [19, 20, 25]—our study minimized potential confounding effects in the analysis of biomechanical parameters and improved the accuracy of assessments. Significant changes in biomechanical parameters were observed in both groups during the 12-month treatment period. The SSI decreased significantly (*P* < 0.05) after 12 months in both groups. Introduced by Eliasy et al. [26], SSI quantifies the corneal material properties in vivo using a finite element algorithm. Previous studies have shown a link between material stiffness and collagen fibril distribution [27–29], thus SSI may indirectly assess the overall arrangement of corneal collagen fibrils. In clinical practice, a reduction in SSI suggests that long-term ortho-k lens wear may reduce corneal material stiffness, reflecting enhanced corneal flexibility and plasticity, an enhanced capacity of the cornea to maintain its deformed state after external forces [30], potentially facilitating the cornea to maintain its remodeled morphology after ortho-k wear. We speculate that these biomechanical alterations may be attributable to the ortho-k-induced rearrangement of corneal collagen fibrils. However, previous in vitro studies in rabbits and monkeys have not demonstrated significant alterations in the diameter or arrangement of corneal stromal collagen fibrils after ortho-k [31, 32]. Future in vivo studies using advanced imaging techniques are warranted to elucidate the microstructural basis of these biomechanical effects.

Our study also revealed increased A1 time and integrated radius, accompanied by decreased A1 velocity, in both BOZD groups after 12 months compared with baseline (*P* < 0.05). The A1 time and velocity represent the time and velocity at which the cornea reaches its first flattening after an air puff, reflecting resistance to deformation and elasticity [33]. The integrated radius represents the area of the inverse concave radius during the concave phase [34]. A longer A1 time, slower velocity to first applanation, and larger integrated radius indicate enhanced corneal resistance to external forces and increased elasticity. These findings may be associated with reduced corneal hydration during ortho-k wear. Alharbi et al. [35] reported that overnight ortho-k exerts positive pressure on the central cornea, which suppresses normal stromal edema during sleep. The combined “clamping effect” of lens and IOP limits central stromal swelling, resulting in markedly lower edema than non-lens-wearing eyes. As the stroma primarily determines the corneal biomechanics, reduced stromal hydration may increase the elastic modulus [36], thereby enhancing the resistance to deformation observed clinically.

**Fig. 4:**
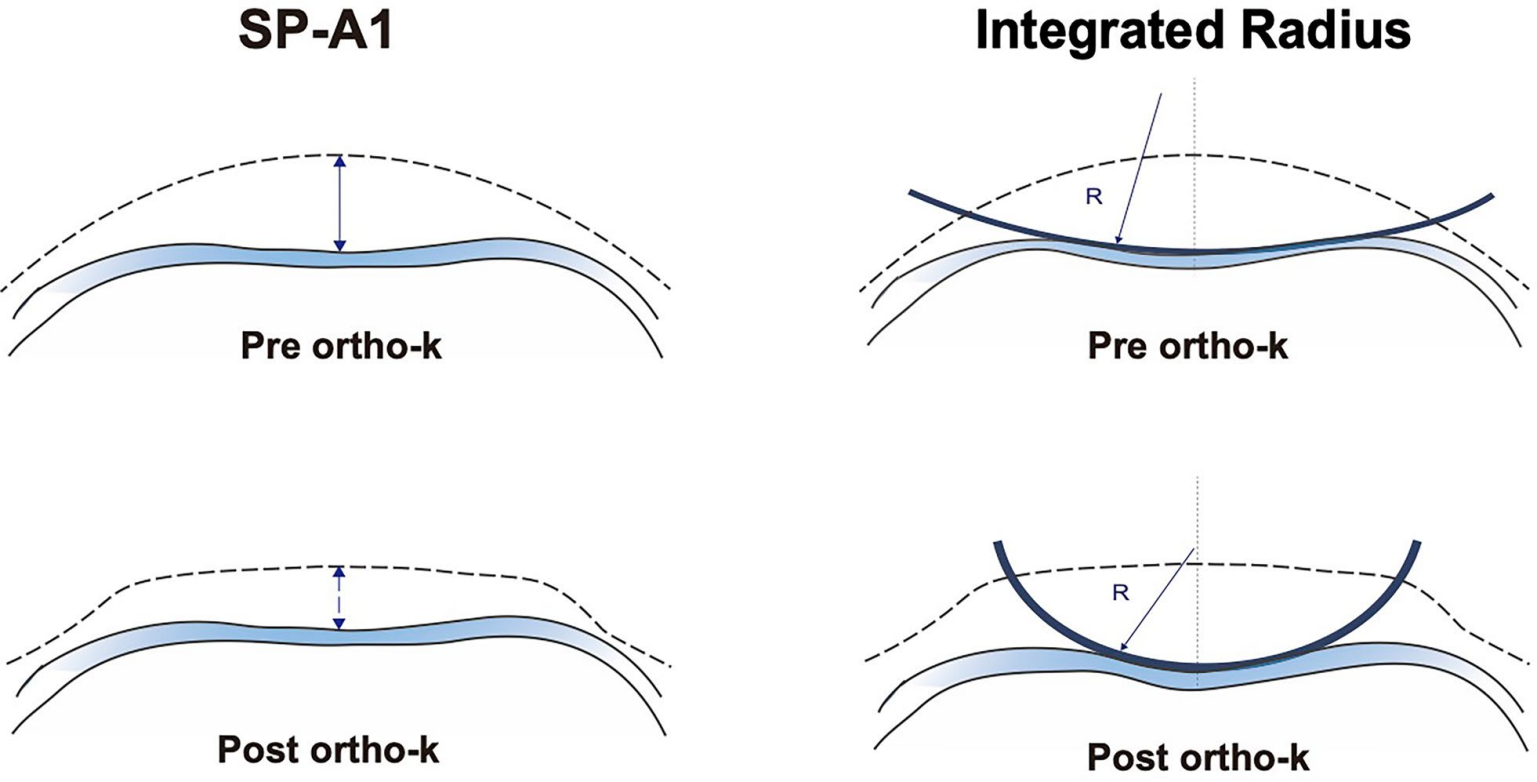
The stiffness parameter at first applanation (SP-A1) and integrated radius before and after orthokeratology (ortho-k) therapy. Ortho-k therapy results in central corneal flattening, which may reduce the displacement required to reach first applanation and produce a small radius of concavity generated under the same air puff

SP-A1 values were significantly elevated from 1-day to 6-month visits in two groups (*P* < 0.05). Developed by Roberts et al. [37] using finite element modeling, SP-A1 represents the corneal stiffness in vivo. Higher SP-A1 values indicate stiffer corneas, a finding consistent with that of Zhang et al. [20], which supports the reliability of our study. SP-A1 was defined as the difference between the air pressure on the corneal surface and the biomechanically corrected IOP divided by the corneal displacement from the initial state to the first applanation. Greater corneal stiffness stabilizes ocular structures and contributes to myopia control [38, 39]. Therefore, ortho-k-induced increases in corneal plasticity and stiffness may act synergistically to enhance myopia control, providing a new biomechanical perspective on the underlying mechanisms. In addition, as shown in Fig. 4, central corneal flattening after ortho-k-induced remodeling may explain the increased SP-A1 and integrated radius, which likely resulted from the reduced displacement to reach applanation and a small radius of concavity generated under the same air puff. The reduction in SSI may reflect collagen fiber reorganization associated with corneal remodeling, whereas the increase in A1 time may be related to intrinsic corneal biomechanical alterations induced by ortho-k.

In this study, ΔAL was significantly smaller in the 5BOZD group than that in the 6BOZD group at both 6 and 12 months (*P* < 0.05), confirming the superior long-term myopia control effect of smaller lenses [11, 12]. Additionally, ΔAL was significantly and positively correlated with ΔA1 time in the 6BOZD group at both intervals (r_6m_ = 0.385; r_12m_ = 0.424; *P* < 0.05), despite being a weak and a moderate correlation, suggesting that corneal biomechanics may be one of the contributing factors to the effectiveness of ortho-k in slowing AL elongation. A1 time is a widely used parameter for diagnosing keratoconus and assessing its biomechanical properties after refractive surgery [40]. Liu et al. [41] pointed out that the A1 time can reflect corneal elasticity, and the greater the myopia severity, the longer the A1 time, indicating greater corneal elasticity. Elasticity refers to the ability of a material to recover its original form after deformation under an external stress [42]. Furthermore, ΔHC time was the parameter with the strongest correlation with ΔAL in the 6BOZD group (r_6m_ = − 0.453; *P* < 0.05). HC time represents the time from the initial state to the highest concavity after an air puff at the corneal apex, with longer times indicating a stiffer cornea. Despite a moderate correlation, our findings indicate that greater AL elongation is associated with shorter HC times in myopic eyes, consistent with the findings of Sun et al. [43]. The A1 time increased, and the HC time decreased dynamically in both groups during follow-up, suggesting an enhanced rebound capacity of the cornea. However, this may also suggest faster recovery after lens removal of the reshaped cornea. The 5BOZD lens produced greater central corneal displacement changes [17], may potentially lead to slower recovery and consequently stronger myopia control. Moreover, smaller BOZDs may offset this corneal biochemical change through optical mechanisms such as enhanced peripheral myopic defocus stimuli to the retina, further limiting AL elongation [14, 15]. Notably, binocular interactions may represent a potential factor influencing AL elongation and require further evaluation in future studies. Our findings provide an experimental basis for individualized ortho-k lens selection in clinical practice. Therefore, patients with rapid disease progression may benefit more from a smaller BOZD. This study offers a new biomechanical perspective for explaining the differences in myopia control efficacy between lenses with different BOZDs.

Corneal biomechanical parameters were not significantly different between the two groups at any follow-up (*P* > 0.05), suggesting that both BOZDs exerted similar and modest long-term effects on corneal biomechanics. However, Zhang et al. [17], using finite element analysis, reported that 5BOZD lenses generated a greater maximum contact pressure over a smaller contact area than 6BOZD lenses. This resulted in a faster change in the central corneal shape. These inconsistencies may have resulted from methodological differences. Most of the corneal biomechanical parameters analyzed in this study were derived from Scheimpflug-based Corvis ST imaging, which may not capture global and anisotropic biomechanical variations.

This study had several limitations. First, because Corvis ST parameters represent the overall corneal biomechanics, subtle localized biomechanical variations induced by ortho-k lenses with different BOZD may not have been accurately characterized. Future studies should employ regional corneal assessment tools such as Brillouin microscopy or optical coherence elastography to map specific spatial variations in corneal stiffness. Second, since the participants were children, discontinuation of ortho-k lens wear could have caused AL to rebound [44, 45], conflicting with the ethical standards of myopia control. Consequently, this study focused solely on biomechanical changes during continuous lens wear. After the 1-year follow-up visit, all participants were given 5BOZD ortho-K lenses in both eyes to minimize interocular differences. Third, given the limited sample size, variability across parameters, and some significant correlations that were not strong, the results must be interpreted with caution. Finally, as this self-controlled study included only Chinese adolescents, there is a potential selection bias in the population. Multi-ethnic crossover studies with larger samples are needed to confirm these findings and avoid potential anisometropia.

## Conclusion

Long-term ortho-k wear resulted in significant increases in SP-A1, A1 time, and integrated radius, along with significant decreases in A1 velocity and SSI, suggesting enhanced corneal biomechanical stability and plasticity. Strengthened corneal biomechanics may be a key factor in the control of AL elongation by ortho-k. The traditional 6BOZD lens induced relatively greater intrinsic changes in corneal elasticity, which may be one of the contributing factors related to its inferior myopia control than the 5-mm design. These findings provide further insight into the biomechanical basis of ortho-k-mediated myopia management and suggest that its different efficacies may reflect optic-mechano-biological coupling. Further histological and in vivo studies are required to elucidate these underlying mechanisms.

## Supplementary Information

Supplementary Material 1

Supplementary Material 2

## Data Availability

This study's supporting data can be obtained by the corresponding author upon reasonable request.

## Abbreviations

AL: Axial length
BOZD: Back optic zone diameter
CCT: Central corneal thickness
GEEs: Generalized estimating equations
IOP: Intraocular pressure
Kf: Flattest keratometry
Ks: Steepest keratometry
ortho-k: Orthokeratology
SP-A1: Stiffness parameter at first applanation
SSI: Stress–strain index
