# Supplementary material for: Effects of orthokeratology with different back optic zone diameters on corneal biomechanics and myopia control: a 1-year randomized, double-blind, self-controlled study
Source: Eye Vis (Lond). 2026 Jul 21;13:31. doi: 10.1186/s40662-026-00503-2 (PMC13386758; doi:10.1186/s40662-026-00503-2)
Supplement: Supplementary file 1 — Supplementary Material 1 [file 40662_2026_503_MOESM1_ESM.docx]

**Additional file 1. Biomechanical parameters provided by the Corvis ST**

| **Parameters** | **Definition** |
| --- | --- |
| A1 and A2 time | Time to reach the first and second applanation |
| HC Time | Time to reach the highest concavity |
| A1 and A2 velocity | Velocity at the first and second applanation |
| DA ratio max (1 mm/2 mm) | Corneal deformation ratio between the corneal apex and corneal apex within 1 mm/2 mm |
| Radius | Radius of curvature at highest concavity |
| Peak distance | Distance between corneal peaks at highest concavity |
| Def. Amp. Max | Deformation amplitude at the highest concavity |
| ARTh | Ambrósio relational thickness to the horizontal profile |
| Integrated radius | Calculus of the radius of the reverse concavity |
| SP-A1 | Stiffness parameter at first applanation |
| SSI | Stress–strain index |
