# Supplementary material for: Effects of orthokeratology with different back optic zone diameters on corneal biomechanics and myopia control: a 1-year randomized, double-blind, self-controlled study
Source: Eye Vis (Lond). 2026 Jul 21;13:31. doi: 10.1186/s40662-026-00503-2 (PMC13386758; doi:10.1186/s40662-026-00503-2)
Supplement: Supplementary file 2 — Supplementary Material 2 [file 40662_2026_503_MOESM2_ESM.docx]

**Additional file 2. Effect sizes of the Corvis ST parameters in the 5BOZD group and 6BOZD groups**

| **Parameters** |  | **Group** | **Effect size** | **SE** |
| --- | --- | --- | --- | --- |
| Def. Amp. Max |  | 5BOZD | −0.026^c^ | 0.012^c^ |
|  |  | 6BOZD | −0.033^c^ | 0.013^c^ |
| A1 time |  | 5BOZD | 0.014^d^  0.013^e^ | 0.007^d^  0.006^e^ |
|  |  | 6BOZD | 0.017^e^ | 0.008^e^ |
| A1 velocity |  | 5BOZD | −0.006^b^  −0.005^c^  −0.005^e^ | 0.002^b^  0.003^c^  0.002^e^ |
|  |  | 6BOZD | −0.008^c^  −0.005^d^  −0.008^e^ | 0.002^c^  0.002^d^  0.002^e^ |
| Peak dist. |  | 5BOZD | −0.064^b^  −0.083^c^ | 0.030^b^  0.029^c^ |
|  |  | 6BOZD | −0.069^d^ | 0.031^d^ |
| ARTh |  | 5BOZD | −64.846^a^  −113.523^b^  −101.520^c^  −100.996^d^  −106.330^e^ | 10.898^a^  17.789^b^  18.509^c^  18.884^d^  15.428^e^ |
|  |  | 6BOZD | −67.736^a^  −102.480^b^  −114.244^c^  −107.642^d^  −101.795^e^ | 13.294^a^  13.470^b^  14.541^c^  12.287^d^  18.660^e^ |
| Integrated radius |  | 5BOZD | 0.305^e^ | 0.127^e^ |
|  |  | 6BOZD | 0.494^e^ | 0.160^e^ |
| SP-A1 |  | 5BOZD | 4.082^a^  8.269^b^  7.859^c^  6.039^d^ | 1.288^a^  1.824^b^  1.877^c^  1.837^d^ |
|  |  | 6BOZD | 4.951^a^  8.592^b^  10.364^c^  8.72^d^  6.97^e^ | 1.656^a^  1.625^b^  1.670^c^  1.51^d^  1.69^e^ |
| SSI |  | 5BOZD | −0.029^e^ | 0.014^e^ |
|  |  | 6BOZD | −0.049^e^ | 0.022^e^ |

GEE models estimate the effect size using the standard error (SE) and the respective *P* value. Def. Amp. Max = the deformation amplitude at the highest concavity; A1 time = the first applanation time; A1 velocity = the first applanation velocity; A2 time = the second applanation time; A2 velocity = the second applanation velocity; HC time = the highest concavity time; Peak dist. = peak distance; DA ratio max 1 mm = corneal deformation ratio between the corneal apex and corneal apex within 1 mm; DA ratio max 2 mm = corneal deformation ratio between the corneal apex and corneal apex within 2 mm; ARTh = Ambrósio relational thickness to the horizontal profile; SP-A1= the stiffness parameter at first applanation; SSI = the stress–strain index.

^a^Statistically significant difference between baseline and 1-day visit.

^b^Statistically significant difference between baseline and 7-day visit.

^c^Statistically significant difference between baseline and 1-month visit.

^d^Statistically significant difference between baseline and 6-month visit.

^e^Statistically significant difference between baseline and 12-month visit.
